# Early‐Life Predictors of Obesity and High Blood Pressure Comorbidity at Midlife: Findings From the 1958 National Child Development Study (NCDS)

**DOI:** 10.1155/jobe/3913006

**Published:** 2026-10-25

**Authors:** Sebastian Stannard, Rhiannon K. Owen, Ann Berrington, Nida Ziauddeen, Simon D. S. Fraser, Shantini Paranjothy, Rebecca J. Hoyle, Nisreen A. Alwan

**Affiliations:** ^1^ School of Primary Care, Population Sciences, and Medical Education Faculty of Medicine University of Southampton Southampton UK southampton.ac.uk; ^2^ Population Data Science Faculty of Medicine, Health, and Life Science Swansea University Medical School Swansea University Swansea UK swansea.ac.uk; ^3^ School of Economic, Social, and Political Sciences University of Southampton Southampton UK southampton.ac.uk; ^4^ School of Medicine, Medical Sciences, and Nutrition University of Aberdeen Aberdeen UK abdn.ac.uk; ^5^ School of Mathematical Sciences University of Southampton Southampton UK southampton.ac.uk; ^6^ University Hospital Southampton NHS Foundation Trust Southampton UK nhs.uk; ^7^ NIHR Applied Research Collaboration Wessex Southampton UK

## Abstract

**Background:**

Early‐life exposures can increase the risk of both obesity and high blood pressure in adulthood. In this paper, we identify exposures across five pre‐hypothesised childhood domains, explore them as predictors of obesity and high blood pressure comorbidity using the 1958 National Child Development Study (NCDS), and discuss these results in comparison to a similar approach using another birth cohort (1970 British Cohort Study (BCS70)).

**Methods:**

The analytical sample included 9150 participants. The outcome was obesity (BMI of ≥ 30) and high blood pressure (blood pressure > 140/90 mm Hg) comorbidity at age 44. Domains included: ‘prenatal, antenatal, neonatal and birth’, ‘developmental attributes and behaviour’, ‘education and academic ability’, ‘socioeconomic factors’ and ‘parental and family environment’. Stepwise backward elimination selected variables for inclusion for each domain, and predicted risk scores of obesity–high blood pressure for each cohort member within each domain were calculated. We performed multivariable logistic regression analysis including domain‐specific risk scores, sex and ethnicity to assess how well the outcome could be predicted taking all domains into account. In additional analysis, we included potential adult factors.

**Results:**

Including all domain‐specific risk scores, sex and ethnicity in the same prediction model the area under the curve was 0.70 (95% CI 0.67–0.72). The strongest domain predictor was the socioeconomic factors domain (OR 1.28 95% CI 1.19–1.38), similar to the BCS70 results. The parental and family environment domain was not a significant predictor (OR 1.08 95% CI 0.94–1.24) unlike the BCS70 results. After considering adult predictors, robust associations remained to the socioeconomic, education and academic abilities, development and behaviour, and prenatal, antenatal, neonatal and birth domains.

**Conclusions:**

In the NCDS, some early‐life course domains were significant predictors of obesity–high blood pressure comorbidity, supporting previous findings. Shared early‐life characteristics could have a role in predicting obesity–high blood pressure comorbidity, particularly for those who faced socioeconomic disadvantage.

## 1. Introduction

In England, an estimated 26% of adults have obesity (body mass index 30 kg/m^2^ or over) [1] and 31% of adults have hypertension also known as high blood pressure (blood pressure over 140/90 mm Hg) [2]. Both conditions are associated with further morbidities including Type 2 diabetes, heart disease, kidney disease, renal disease, strokes and some cancers, including breast and bowel cancer [3–6]. Both conditions are also strongly associated with all‐cause mortality [7, 8].

Globally, both conditions represent a major public health problem. The World Health Organisation (WHO) estimates that 2.8 million deaths every year are attributed to overweight or obesity [9]. Hypertension prevalence has increased between 41% and 144% across WHO regions over the past 30 years [10]. Research has also demonstrated that obesity and hypertension often co‐occur [11, 12]. For example, around half of hypertensive patients in the United States have obesity [13], and individuals with obesity are 3.5 times more likely to have hypertension compared to normal weight individuals [14, 15]. The co‐occurrence of both conditions also increases the risk of further morbidity including cardiovascular disease, sexual dysfunction, poor quality of life and mortality [15, 16].

It is important to consider risk exposures to morbidities such as obesity and hypertension in terms of multiple domains (i.e., a group of variables that represent an overarching theme) for three reasons. First, this provides a combined exposure measure that reflects multiple variables in the data rather than performing multiple statistical testing using single components in relation to the study outcomes. Second, conceptualising the components within wider early‐life domains provides a more holistic reflection of the childhood conditions in which children grew up. Third, considering early‐life risk in domains may better inform interventions and policy in childhood as incorporating information from multiple early‐life domains into the same analysis may help us understand the combined effects of different experiences across a range of early‐life domains on developing long‐term conditions. This can provide actionable insights into developing interventions in childhood that may help support people to live longer in good health.

In previous research [17], we developed a conceptual framework to characterise the population‐level domains of early‐life determinants of future multimorbidity risk. Through a scoping literature and policy review, and with the support of public and patient contributors, 12 domains of early‐life risk factors of future multimorbidity risk were identified [17]. These domains covered a range of social, economic, developmental, educational and environmental factors that focused on both direct and indirect factors as well as wider systemic and structural determinants of disease and health inequalities [17, 18]. Subsequent research [19] explored how these 12 domains of early‐life risk factors of future multimorbidity risk could be characterised across U.K. birth cohort studies including the 1958 National Child Development Study (NCDS).

In a previous paper, we used the 1970 British Cohort Study (BCS70) to consider five early‐life domains, chosen because they showed unadjusted associations with obesity and high blood pressure comorbidity at age 46 [20]. Results indicated that all five domains were significant predictors for obesity–high blood pressure comorbidity, with the strongest domain predictors being the parental and family environment domain and the socioeconomic factors domain [20]. After including adult predictors, the most robust domains for predicting obesity–high blood pressure included the parental and family environment domain, socioeconomic factors domain, and education and academic ability [20].

In this analysis, we aimed to apply the same methodological approach to the NCDS cohort. Although the two cohorts are only 12 years apart, the childhood conditions the members of each cohort grew up in are arguably different. The younger cohort (BCS70) experienced rising women’s employment and economic independence, decreased family stability, educational expansion and a shift away from the male breadwinner nuclear family that dominated the family structure of the NCDS cohort [21–25]. The younger cohort (BCS70) also experienced generational pay progression, higher real household disposable incomes and an increase in home ownerships [21], but globalisation and a shift of income from labour to capital meant greater economic uncertainty and increased inequality [25]. Therefore, these factors might lead us to expect more heterogeneity in the early‐life experiences of the 1970 cohort compared to the 1958 cohort.

In this study, we aimed to explore the extent to which the same five early‐life course domains predict the outcome of obesity–high blood pressure comorbidity. To achieve this while weighting the components of each domain, we also produced—as a first stage of the analysis—predicted risk scores of the outcome for each of the five pre‐defined early‐life domains. In the discussions, we consider the results presented here in relation to a previous analysis of the BCS70 cohort [20]. This work forms part of a larger aim to model targeted multimorbidity prevention scenarios as part of the Multidisciplinary Ecosystem to study Lifecourse Determinants and Prevention of Early‐onset Burdensome Multimorbidity (MELD‐B) project [26].

## 2. Methods

### 2.1. Datasets

We used the 1958 NCDS [27] that has followed children born in England, Scotland and Wales in one week in 1958, and includes 17,415 cohort members. The comorbidity outcome of obesity and high blood pressure was measured in a biomedical sweep at age 44. All other variables were measured either at birth or at the age 11 sweep.

### 2.2. Outcome

The outcome was a combined obesity–high blood pressure phenotype at age 44. Blood pressure was measured using an automated Omron sphygmomanometer according to a standardised protocol. Three readings were obtained from the left arm at 1‐min intervals, with cuff size selected based on arm circumference, during a single appointment from a research nurse. High blood pressure was defined as an average systolic blood pressure ≥ 140 mmHg and/or an average diastolic blood pressure ≥ 90 mmHg. BMI was calculated via height and weight measurements taken during the same nurse appointment, and calculated using the following formula: BMI = weight (kg)/height (m)^2^. Obesity was defined as a BMI of 30 kg/m^2^ or over. Height was measured without shoes using a portable stadiometer and recorded to the nearest millimetre. Weight was measured using calibrated Tanita electronic scales, with participants asked to remove shoes and heavy outer clothing before measurement. The obesity–high blood pressure comorbidity variable was considered as a binary (no/yes) variable. Therefore, in our sample, participants who were classified as ‘yes’ had both obesity and hypertension, whereas ‘no’ included everyone else.

### 2.3. Exposures (Five Pre‐Hypothesised Domains)

We focus on five domains because they showed unadjusted association with the outcome in this paper and in the previous research based on the BCS70 cohort [20], and Supporting Information Table 1 includes all the variables that were considered for each domain:

1. Prenatal, antenatal, neonatal and birth domain focused on the period from conception to the onset of labour, the circumstances and outcomes surrounding a birth, and the period immediately following birth.
2. Developmental attributes and behaviour domain focused on the developmental markers of children relating to cognition, coordination, personality types and behavioural traits.
3. Child education and academic ability domain related to the process of learning and educational achievement, especially in educational settings, and the knowledge an individual gains from these educational institutions.
4. Socioeconomic factors domain included factors relating to differences between individuals or groups of people caused mainly by their social and economic situation.
5. Parental and family environment domain incorporated the interactions between children and care givers, parenting styles, parental beliefs, attitudes and discipline, and wider family factors such as kin networks.

### 2.4. Analytical Sample

The analytical sample included all cohort members who had measured height, weight and blood pressure at age 44 (*n* = 9150/17,415). The proportion of missing data for all variables included in the analytical sample are presented in Supporting Information Table 2, missingness ranged from approximately 5% to 19% across variables. Multiple imputation was used on the sample present at age 44 (*n* = 9150) to maintain sample size and reduce bias in the estimates due to missing data. Multiple imputation was conducted by chained equations for missing observations at birth and age 11 and 44 [28]. Fifty imputation cycles were constructed under the missing‐at‐random assumption [29–31], which has been found to be highly plausible in the British birth cohorts [32]. All variables were included in the imputation process. The outcome was included in the imputed models, but imputed outcome values were not used.

### 2.5. Statistical Analysis

We used the same methodological approach followed to examine five early‐life domains as predictors of obesity–high blood pressure comorbidity in the BCS70 cohort, and full details of this method can be found elsewhere [20]. However, we provide a summary of those methods here.

First, stepwise backwards elimination was conducted separately within each of the 50 imputed datasets for each domain, using a retention threshold of *p* = 0.157 [20]. Variables retained in more than half of the 50 imputed‐dataset models were included in the final variable set for that domain. This common set of selected variables was then used to fit the domain‐specific models across all imputed datasets.

Second, logistic regression models then explored the relationship between retained variables following stepwise backwards elimination and odds of obesity–high blood pressure comorbidity. From this regression modelling, the predicted risk scores of the obesity–high blood pressure comorbidity outcome for each cohort member within each domain were then calculated.

Third, we used logistic regression modelling including all five domain‐specific risk scores, and added sex (measured at birth), and ethnicity (White British or Other) to predict the obesity–high blood pressure comorbidity outcome. The area under curve (AUC) statistic was used to assess the predictive performance of this model. The odds ratios of the five domains within this model were used to identify the strongest domains that acted as predictors for obesity and high blood pressure comorbidity taking into account the effect of the other domains. These domain‐specific risk scores were model‐derived predicted values and were entered into subsequent regression models as continuous predictors. As such, their scale reflects the underlying prediction model within each domain and does not correspond to a directly interpretable unit of measurement.

Finally, we repeated the previous step this time with the inclusion of adult factors that are potentially linked to both the exposures and the outcome. These were recorded at age 44 and included marital status, self‐rated financial difficulty, the age the cohort member left education, smoking status, frequency of consuming alcoholic drinks, frequency of consuming fresh fruit, frequency of exercising and occupational social class.

Discrimination was assessed using the pooled AUC, and calibration was assessed using the pooled calibration slope. No bootstrap resampling, cross‐validation or split‐sample internal validation was performed. The reported measures therefore represent apparent model performance and should be interpreted with caution.

### 2.6. Ethical Approval

Ethics approval for MELD‐B was obtained from the University of Southampton Faculty of Medicine Ethics Committee (ERGO II Reference 66810). A full review of ethics and consent for the NCDS study is provided by the Centre for Longitudinal Studies and can be found elsewhere [33].

## 3. Results

### 3.1. Step 1: Stepwise Backwards Elimination to Select Variables for Inclusion Separately for Each Domain

Table 1 identifies the retained variables following stepwise backwards elimination for each domain in relation to the outcome of obesity–high blood pressure comorbidity at age 44. Amongst the 9150 cohort members at age 44, 4% (*n* = 395) had obesity–high blood pressure comorbidity. Considering the individual prevalence, 24% of the cohort members at age 44 had obesity (*n* = 2235/9150) and 11% had high blood pressure (*n* = 1049/9150). For the prenatal, antenatal, neonatal and birth domain, the retained variables following stepwise backwards elimination focused on the health of the cohort member at birth (illness, resuscitation and admission to hospital). For the developmental attributes and behaviour domain, retained variables considered developmental markers of the cohort members (coordination and balance), whilst father’s social class was retained within the socioeconomic factors domain. For the child education and academic ability domain, retained variables were related to academic ability (general ability test, general knowledge assessment and oral ability assessment), and for the parental and family environment domain, retained variables focused on fathers’ interest in their child’s education and engaging in family activities (family walks).

**TABLE 1:** Step 1: variables retained in the five domain risk scores following stepwise backwards elimination, and prevalence of combined obesity–high blood pressure at age 44 (raw, unimputed data).

			Obesity and high blood pressure		
			No *N* (%)	Yes *N* (%)	Total sample^1^
Prenatal, antenatal, neonatal and birth domain (variables recorded at birth)	Child resuscitation at birth	No	7934 95.7%	357 4.3%	8291
		Yes	158 92.4%	13 7.6%	171
	Hospital admission at birth	No admission	6549 96.0%	273 4.0%	6833
		Admission for toxaemia	562 92.7%	55 7.2%	606
		Admission for other condition(s)	975 95.3%	48 4.7%	1023
	Baby illness at birth	No illness	7840 95.7%	352 4.3%	8192
		Illness	207 92.8%	16 7.2%	223

Developmental attributes and behaviour domain (variables recorded at age 11)	Poor physical coordination	Not at all	6311 95.7%	284 4.3%	6595
		Somewhat	870 94.8%	48 5.2%	918
		Certainly applies	134 93.1%	10 6.9%	144
	Standing heel to toe	Very steady	3807 96.2%	150 3.8%	3957
		Slightly unsteady	2667 95.1%	140 5.0%	2807
		Very unsteady	587 94.1%	37 5.9%	624

Socioeconomic factors domain (variables recorded at age 11)	Fathers social class	I	460 97.7%	11 2.3%	471
		II	1451 97.5%	39 2.6%	1490
		III (non‐manual)	742 96.4%	28 3.6%	770
		III (manual)	3026 95.0%	159 5.0%	3185
		IV (non‐manual)	138 92.0%	12 8.0%	150
		IV (manual)	1085 95.1%	56 4.9%	1141
		V	359 92.3%	30 7.7%	389

Child education and academic ability domain (variables recorded at age 11)	General knowledge—teachers assessment	Exceptional	2209 96.4%	82 3.6%	2291
		Above average	3456 95.3%	170 4.7%	3626
		Average and below	1880 95.1%	97 4.9%	1977
	Oral ability—teachers assessment	Exceptional	1995 97.1%	60 2.9%	2055
		Above average	4214 95.3%	208 4.7%	4422
		Average and below	1336 94.3%	81 5.7%	1417
	General ability test score—mean (SD)		45.7 (15.3) N = 7587	42.0 (15.7) N = 347	

Parental and family environment domain (variables recorded at age 11)	Family walks	Most weeks	4057 96.1%	172 3.9%	4229
		Occasionally	2966 95.2%	150 4.8%	3116
		Hardly ever	386 93.5%	27 6.5%	413
	Father’s interest in child’s education	Very interested	3232 96.3%	124 3.7%	3356
		Some interest	2539 95.0%	134 5.0%	2673
		Little interest	862 94.7%	48 5.3%	910
		Can’t say/no father	780 95.2%	39 4.8%	819

^1^The total sample size was based on the specific sample available at the sweep in which the variable was reported or measured.

### 3.2. Step 2: Predicted Risk Scores of Obesity–High Blood Pressure Comorbidity for Each Cohort Member Within Each Domain

In Supporting Information Tables 4–8, we include the regression coefficients of obesity–high blood pressure for the logistic regression models that explored the relationship between retained variables following stepwise backwards elimination (step 1) and obesity–high blood pressure comorbidity, and for each domain separately. In Supporting Information Table 3, we present a Pearson correlation matrix exploring correlation across domains. The strongest correlations were between the parental and family environment domain and child education and academic ability domain (coefficient 0.31) and between the parental and family environment domain and socioeconomic factors domain (coefficient 0.29). These results suggest that the domain‐specific risk scores were not collinear.

### 3.3. Step 3: A Prediction Model Including Five Domain‐Specific Risk Scores, Sex and Ethnicity

The AUC for the prediction model that included all five domain‐specific risk scores (produced in step 2), ethnicity and sex was 0.70 (95% CI 0.67–0.72); for reference, the AUC with just ethnicity and sex was 0.63 (95% CI 0.60–0.65). The model demonstrated good calibration, with a pooled calibration slope of 0.98 (SE = 0.07). Table 2 presents the odds ratios of obesity–high blood pressure at age 44 for the prediction model that included all five domain‐specific risk scores (produced in step 2), sex and ethnicity. As shown, the parental and family environment domain was not a significant predictor of obesity–high blood pressure comorbidity (OR 1.08 95% CI 0.94–1.24). All other domains were predictors of obesity–high blood pressure comorbidity, with the strongest domain predictor being the socioeconomic factors domain (OR 1.28 95% CI 1.19–1.38).

**TABLE 2:** Step 3: odds ratios of obesity–high blood pressure at age 44 in relation to domain‐specific risk score of obesity–high blood pressure for five early‐life domains.

	Odds ratios	95% confidence interval
Developmental attributes and behaviour domain	**1.20**	**1.06–1.36**
Prenatal, antenatal, neonatal and birth domain	**1.21**	**1.11–1.31**
Parent and family environment domain	1.08	0.94–1.24
Education and academic ability domain	**1.12**	**1.02–1.23**
Socioeconomic factors domain	**1.28**	**1.19–1.38**
Child’s sex: women (base—men)	**0.78**	**0.64–0.91**
Mother ethnic group: other (base—White)	1.37	0.86–2.21
Number of observations		9150

*Note:* Multiple imputed data (50 Imputations). Significant findings highlighted in bold. Domain‐specific risk scores represent model‐derived predicted risk scores and are not standardised *z*‐scores; therefore, odds ratios should be interpreted as relative associations with the score rather than as the effect of a specific measurable unit.

### 3.4. Step 4: A Prediction Model Including Five Domain‐Specific Risk Scores, Sex, Ethnicity and Potential Adult Predictors

Including adult factors in the model, the AUC increased to 0.73 (95% CI: 0.71–0.76), and the model demonstrated good calibration, with a pooled calibration slope of 0.99 (SE = 0.05). Table 3 presents the odds ratios of obesity–high blood pressure at age 44 for this prediction model that included all five domain‐specific risk scores (produced in step 2) ethnicity, sex and adult factors. As shown, after the inclusion of adult factors, the parent and family environment domain was not a significant predictor of obesity–high blood pressure comorbidity (OR 1.08 95% CI 0.94–1.25). All the other four domains remained robust predictors for obesity and high blood pressure comorbidity, with the strongest domain predictor for obesity and high blood pressure comorbidity remaining as the socioeconomic factors domain (OR 1.29 95% CI 1.19–1.39).

**TABLE 3:** Step 4: odds ratios of obesity–high blood pressure at age 44 in relation to domain‐specific risk score of obesity–high blood pressure for five early‐life domains including adult predictors.

	OR	95% confidence interval
Developmental attributes and behaviour domain	**1.19**	**1.05–1.35**
Prenatal, antenatal, neonatal and birth domain	**1.20**	**1.11–1.30**
Parent and family environment domain	1.08	0.94–1.25
Education and academic ability domain	**1.13**	**1.03–1.25**
Socioeconomic factors domain	**1.29**	**1.19–1.39**
Child’s sex: Women (base—men)	**0.42**	**0.32–0.54**
Mother ethnic group: Other (base—White)	1.44	0.88–2.33
Smoking: Used to smoke (base—never smoked)	0.93	0.73–1.19
Occasionally smokes	**0.40**	**0.19–0.83**
Regularly smokes	**0.59**	**0.42–0.76**
Marital status: Cohabiting (base—married)	**0.53**	**0.33–0.84**
Single never married	1.12	0.78–1.60
Separated/divorced	0.77	0.52–1.16
Alcohol consumption: most days (base—occasionally)	**1.67**	**1.10–2.54**
2–3 days a week	1.31	0.88–1.95
Once a week	1.02	0.66–1.57
2–3 times a month	**1.88**	**1.20–2.93**
Never	1.17	0.63–2.14
Social class: lower managerial (base—higher managerial)	1.64	0.89–3.02
Skilled manual	1.34	0.67–2.65
Skilled nonmanual	1.77	0.93–3.37
Partly skilled	1.51	0.74–3.01
Unskilled	1.11	0.43–2.86
Unemployed	1.65	0.80–3.39
Days exercise per week: 4–5 days (base—every day)	1.38	0.88–2.16
2–3 days	1.32	0.91–1.94
Once a week	1.21	0.81–1.80
2–3 times a month	1.60	0.98–2.60
Less often	**1.67**	**1.17–2.38**
Fruit consumption: every day (base—more than once a day)	1.18	0.85–1.61
3–6 days a week	1.31	0.89–1.90
1–2 days a week	1.05	0.72–1.51
Less than once a week	**1.63**	**1.03–2.58**
Occasionally	1.04	0.69–1.57
Never	**1.87**	**1.01–3.44**
Managing financially: doing all right (base—doing well)	0.82	0.64–1.06
Just about getting by	0.96	0.72–1.29
Finding it quite difficult	0.98	0.60–1.62
Finding it very difficult	0.73	0.31–1.72
Age left education	0.97	0.92–1.04
Number of observations		9150

*Note:* Multiple imputed data (50 imputations). Significant findings highlighted in bold.

## 4. Discussion

Using the NCDS birth cohort dataset, and considering five early‐life domains concurrently, the strongest domain predictor for obesity–high blood pressure comorbidity was the socioeconomic factors domain, even after accounting for socioeconomic factors during adulthood. Results suggested that the parental and family environment domain was not a significant predictor for obesity–high blood pressure comorbidity in adulthood. The inclusion of adult factors slightly improved the predictive performance of the models; however, robust associations remained to the development and behaviour, the socioeconomic factors, education and academic abilities and prenatal, antenatal, neonatal and birth domains.

Our aim in this paper was to use the same methodological approach that has already been applied to the BCS70 cohort [20] to explore the relationship between five early‐life domains and obesity–high blood pressure comorbidity, and then compare results across the two cohort studies. We found that the results presented here were largely comparable to our previous findings based on the BCS70 cohort [20]; in particular, there was consistency with regards to the robustness of the socioeconomic factors domain as a predictor for obesity–high blood pressure comorbidity across both papers [20]. Significantly, the socioeconomic factors domain (in childhood) remained an important predictor even after accounting for socioeconomic factors during adulthood. This adds to a body of literature that has found childhood socioeconomic factors to be associated to multimorbidity later in the life course [34–38], and provides further justification for interventions targeting socioeconomic conditions in childhood as this might have the biggest impact on improving certain health outcomes later in the life course. The findings that the socioeconomic domain remains important across cohorts is significant given the BCS70 cohort (born 1970) were exposed to better economic conditions including higher employment rates particularly for women, generational pay progression, higher real household disposable incomes and an increase in home ownerships [21].

The predictive models demonstrated only modest discrimination (AUC 0.70–0.73), which may limit their suitability for individual‐level risk prediction or public health decision‐making. However, the degree of performance is not unexpected given the long interval between early‐life domains and the midlife outcome. Further, our objective was not to develop an operational prediction tool but to utilise prediction modelling methodologies to quantify the extent to which competing early‐life domains contribute to long‐term risk of obesity–high blood pressure comorbidity. A substantial body of research demonstrates that early‐life domains such as low childhood socioeconomic status are associated with poorer health trajectories across the life course [39, 40]. Our findings therefore highlight the importance of these upstream determinants as targets for early‐life preventive interventions and policy measures, even if their long‐term predictive accuracy for specific midlife conditions is limited.

Overall, the AUC statistics suggested that for both models (including and excluding adult predictors), the NCDS cohort analysis had a stronger predictive power than BCS70 cohort analysis (NCDS AUC 0.70–0.73 vs. BCS70 AUC 0.63–0.68 [20]). Odds ratios (across all models) in the statistical analysis of the NCDS cohort were also larger than in the statistical analysis of the BCS70 cohort (NCDS odds ratios ranged between 1.08 and 1.28 vs. 1.06 and 1.11 in the BCS70 [20]). Further, after considering the role of adult predictors, more robust associations between early‐life domains and high blood pressure and obesity comorbidity remained in the statistical analysis of the NCDS cohort compared to the statistical analysis of the BCS70 cohort (NCDS odds ratios ranged between 1.08 and 1.29 vs. 1.05 and 1.11 in the BCS70 [20]). These three factors may indicate that obesity–high blood pressure comorbidity iscould be more closely related to the domain‐specific risk scores in the NCDS compared to the BCS70.

For the statistical analysis of the BCS70 cohort, the parental and family environment domain was a robust predictor of obesity–high blood pressure (OR 1.11 95% CI 1.05–1.17 [20]), but in the NCDS cohort, it was not a predictor. One explanation for this difference is the wider demographic and social context of the parental‐family environment in which each cohort grew up. For the NCDS cohort (born 1958), most children grew up in households dominated by a stable nuclear‐family model [41]. However, the BCS70 cohort (born 1970) grew up during a period of rapid change within the parental‐family environment. The liberalisation of divorce laws coupled with changing attitudes and norms relating to marriage were reflected in a sharp rise in divorce rates and a decline in first marriage rates from the 1970s onwards [42, 43]. The BCS70 cohort grew up during a period of increasing rates of parental partnership dissolution, single‐parent households and a rise in children living with nonbiological parents and nonfull siblings, and this is reflected by the retained variables following stepwise backwards elimination (step 1 of the analysis).Specifically for the statistical analysis of the BCS70, unlike the NCDS, retained variables relating to the father of the cohort member including father figure and if the father helps manage the cohort member. Therefore, it could be hypothesised that the impact of these rapid changes to the parental‐family environment, particularly around the role of fathers, had a greater impact on the BCS70 cohort compared to the NCDS who were less exposed to these changes. This is demonstrated in a paper that found women in the BCS70 who experienced parental separation were more like to have high blood pressure at age 46, compared to those who had not experienced a parental separation in childhood [44].

A further cohort effect is within the antenatal, neonatal, prenatal and birth domain, which was a robust predictor of obesity–high blood pressure comorbidity in statistical analysis of the NCDS cohort (OR 1.20 95% CI 1.11–1.30) but not in the statistical analysis of the BCS70 cohort (OR 1.05 95% CI 0.99–1.11 [20]). It is likely that even over this short 12‐year period, obstetric, neonatal and speciality care for ill infants will have improved [45], thus potentially reducing the impact of antenatal, neonatal, prenatal and birth adversity in the younger cohort (BCS70). Another difference between the antenatal, neonatal, prenatal and birth domain was that the stepwise backwards selection (step 1 of the analysis) retained different variables. The variables retained in the NCDS cohort focused on the health of the child at birth, whereas, for the BCS70 cohort, the retained variables focussed on factors relating to maternal fertility history and maternal health behaviours during pregnancy.

In the NCDS, the prevalence of obesity, high blood pressure and combined obesity–high blood pressure was lower than that observed in the BCS70 cohort (4.0% vs. 7.6% [20]), suggesting that those with the outcome in the NCDS cohort may be a more select group of individuals. However, given that blood pressure increases with age [46] and that the BCS70 cohort were slightly older than the NCDS cohort at outcome data collection, it is important to note that this relationship between blood pressure and age might explain some of the increased prevalence of obesity–high blood pressure amongst the older BCS70 cohort. In the BCS70 study, we additionally classified high blood pressure if a participant had received a doctor’s diagnosis of high blood pressure or hypertension at age 46 (self‐reported) even if the blood pressure measurement was less than 140/90 mm Hg, to account for those who may have had a lower blood pressure reading due to hypertension medication. In the NCDS, participants were not asked about medical diagnosis of high blood pressure, so it is likely that some treated hypertensive individuals with controlled blood pressure at the time of measurement at age 44 were misclassified as normotensive. These definitional differences between cohorts may, therefore, have contributed to differences in observed prevalence of high blood pressure between the two cohorts, and should be considered when interpreting comparisons.

We examine whether early‐life exposures across five prespecified domains predicted obesity–high blood pressure comorbidity at midlife, and compared the results to previous findings. The NCDS cohort data did not include measures suitable for evaluating childhood interventions, and our aim was not to assess behavioural mechanisms, or propose specific preventive strategies. Instead, our findings highlight that, when early‐life exposures are considered simultaneously within a domains‐based framework, socioeconomic disadvantage emerges as the most robust predictor of later comorbidity. By modelling multiple early‐life domains together, this approach moves beyond single‐variable analyses to consider which domains retain independent influence once competing childhood domains are accounted for, underscoring the value of structural and early‐life policy approaches.

There are a number of important next steps. We have identified robust domains that predict obesity–high blood pressure comorbidity; the next step therefore could include modelling prevention scenarios within these domains to better inform policy to help people live in better health for longer. Second, it is important to expand the methods presented here to consider the relationship to other multimorbidity clusters and outcomes that develop a more sophisticated understanding of multimorbidity, including focussing on burdensomeness and complexity, for example, incorporating eight themes of ‘work’ burden for those living with multimorbidity [47].

Our study has identified a number of key findings. First, early‐life socioeconomic disadvantage was consistently associated with obesity and high blood pressure comorbidity in both cohorts, even after accounting for other early‐life domains and adult factors. This supports existing evidence that childhood socioeconomic conditions may have long‐term implications for cardiometabolic outcomes. Second, although our study was not designed to formally test cohort differences, the patterns observed suggest that the relative importance of early‐life domains may not be uniform across cohorts. For instance, the parental and family environment domain contributed to prediction in the BCS70 cohort but not in the NCDS, while the antenatal and neonatal domain contributed in the NCDS but not in BCS70. These observations should be interpreted cautiously given differences in measurement and outcome definitions between cohorts. Finally, by analysing multiple early‐life domains simultaneously using domain‐specific risk scores, we illustrate how a domains‐based approach can highlight which types of early‐life exposures remain influential when considered alongside one another and after adjustment for adult circumstances. This provides useful insight into which domains may be most relevant for future life‐course–oriented prevention research.

### 4.1. Strengths and Limitations

The NCDS dataset allowed us to explore a wide array of social, environmental and family variables in childhood to represent five early‐life domains. The data also afforded the opportunity to analyse potential adult predictors and objective measures of both obesity and high blood pressure measured at midlife. However, the NCDS cohort was affected by varying levels of missing data reflecting long‐term cohort attrition and item nonresponse. To minimise the bias that would arise from restricting analyses to complete cases, we applied multiple imputation, which helped to maintain sample size, preserve statistical power and produce less biased estimates under the missing at random assumption [48].

A limitation of the NCDS cohort was the significant under‐representation of individuals from non‐White ethnic backgrounds and therefore the cohort does not reflect the ethnic diversity of the British population today. Further BMI does not distinguish between fat mass and lean mass, it provides no information about where fat is distributed, and it can classify people inaccurately across different ages, sexes and ethnic backgrounds [49, 50]. In some groups, such as older adults or certain ethnicities, BMI can underestimate total body fat, while in more muscular individuals, it may overestimate adiposity. It also offers no insight into diet, physical fitness, metabolic health or organ function, and its links with health outcomes are shaped by numerous confounding variables [51]. Rubino et al. [51] argue that clinical assessment should therefore include additional indicators, such as waist circumference, body fat percentage and functional measures, to more accurately confirm obesity status. These measures were not available in the NCDS dataset. Additionally, due to data constraints, our definition of high blood pressure relied solely on measured blood pressure at age 44. This approach does not capture individuals who may have received a prior diagnosis of high blood pressure and are currently on antihypertensive treatment, resulting in controlled blood pressure at the time of measurement. Consequently, some treated hypertensive individuals may have been misclassified as normotensive.

Further, the analytical sample was broadly representative of the original cohort, although some selective attrition was evident. Compared with the childhood sample, those included at age 44 were slightly less likely to be male (49.8% vs. 51.7%), from ethnic minority backgrounds (4.4% vs. 5.1%), report childhood financial hardship (9.6% vs. 11.2%) or come from the lowest social class backgrounds (5.1% vs. 6.2%). While these differences were relatively small, they suggest that attrition was associated primarily with sex and childhood socioeconomic circumstances, which may have introduced some bias into the estimates.

The NCDS provides rich information on both childhood and adult circumstances. In our analysis, we were able to adjust for several adult factors that are associated with obesity and high blood pressure, including marital status, financial difficulty, age at leaving education, smoking status, alcohol consumption, fresh fruit consumption, frequency of exercise and occupational social class. However, these variables were captured cross‐sectionally in adulthood and do not fully reflect risk trajectories or patterns over the life course. We were unable to include direct measures of sedentary behaviour, despite its well‐established role in obesity and cardiometabolic risk [52]. Some proxy information is partially reflected in the reported frequency of exercise, but this does not capture sedentary time or screen‐based behaviours. Similarly, our dietary measure was limited to fresh fruit consumption and does not represent the complexity of diet quality, energy intake, or changes in food environments over time. It is also likely that childhood socioeconomic disadvantage shapes adult outcomes such as poorer diet, lower levels of physical activity, and greater sedentary time, which in turn may contribute to later obesity and high blood pressure comorbidity. However, we were unable to capture these pathways within our models. Given these limitations, our findings should be interpreted with caution, as focusing solely on socioeconomic disadvantage would risk overlooking some of the broader behavioural and environmental factors through which early‐life conditions exert their influence on obesity–high blood pressure comorbidity [53].

We acknowledge that the use of backward stepwise elimination could introduce risks of model instability, sample dependence and overfitting [54, 55]. Stepwise procedures can also produce biased coefficient estimates [56]. These issues can be exacerbated in multiply imputed datasets and in analyses of rare outcomes, where sampling variability is larger and variable selection becomes particularly unstable. However, as discussed by Royston and Sauerbrei [57], although all variable selection strategies attract criticism, backward elimination offers several practical advantages. Because it begins with the full model, it tends to retain strong predictors, and using a threshold such as *p* = 0.157 provides a good approximation to AIC‐based selection. It has also generally been found to perform better than forward selection procedures, as forward methods allow variables to enter the model only once and can miss important combinations of predictors [57].

Given our methods explored domains that were derived from combined risk factors, as opposed to considering these risk factors individually, there is a possibility that our prediction models are over‐adjusted [58]. However, we felt this was unavoidable given our research question was to consider domains rather than individual risk factors. Further, as we have argued previously, we know that children’s early‐life experiences are intersecting, and therefore research must explore methods that can analyse multiple domains simultaneously, as this represents the best approach to disentangle and understand the role of competing early‐life domains for future health outcomes. In previous research, we considered alternative methods to achieve the aims of this paper such as deriving each variable (within each domain) into a binary (yes/no) outcome, and then summing to produce a count of adversity within each domain [59]. However, this approach was more limited (than the one presented here), given we were required to assume that all variables (within each domain), and all domain risk scores, carried equal weight. Second, by deriving variables into a binary indicator, we disregarded information contained within the original data structure, and for most variables, we had to implement arbitrary binary cut‐off points.

## 5. Conclusions

Our findings suggest that some early‐life course domains may be associated with obesity–high blood pressure comorbidity across two longitudinal cohorts. There were some differences across cohorts for the antenatal, neonatal, prenatal and birth domain and parental and family environment domain; we hypothesise that some of these differences could reflect cohort effects related to the environments in which the cohorts grew up. Overall, shared early‐life characteristics may have a role in the development of obesity–high blood pressure comorbidity, particularly among those who experienced socioeconomic disadvantage in early life. These findings support continued investigation of public health approaches aimed at giving children the best start in life.

## Author Contributions

Sebastian Stannard, Rhiannon K. Owen, Ann Berrington, Simon D. S. Fraser, Shantini Paranjothy, Rebecca J. Hoyle and Nisreen A. Alwan contributed to the conceptualisation of the MELD‐B project. Sebastian Stannard, Simon D. S. Fraser and Nisreen A. Alwan obtained the datasets. All authors contributed to the conceptualisation of the paper. Sebastian Stannard and Nisreen A. Alwan led the design and planning of the paper. Rhiannon K. Owen led the design of the statistical analysis. Sebastian Stannard, Rhiannon K. Owen, Ann Berrington, Nida Ziauddeen, Rebecca J. Hoyle and Nisreen A. Alwan supported the design, planning and reviewing of the statistical analysis. Sebastian Stannard performed the statistical analysis with support from Nida Ziauddeen. Sebastian Stannard prepared all figures and graphs. Sebastian Stannard and Nisreen A. Alwan produced the initial draft of the manuscript. All authors were involved in editing and reviewing the manuscript. Sebastian Stannard, Simon D. S. Fraser and Nisreen A. Alwan take responsibility for the data and research governance.

## Funding

This work was supported by National Institute for Health and Care Research, 10.13039/501100000272, NIHR203988.

## Disclosure

All authors approved the final manuscript. This paper has been presented as a preprint on MedRxiv, and a copy of the preprint can be found here: https://www.medrxiv.org/content/10.1101/2024.12.09.24318705v1.full.

## Conflicts of Interest

Rhiannon K. Owen is a member of the National Institute for Health and Care Excellence (NICE) Technology Appraisal Committee, member of the NICE Decision Support Unit (DSU) and associate member of the NICE Technical Support Unit (TSU). She has served as a paid consultant to the pharmaceutical industry and international reimbursement agencies, providing unrelated methodological advice. She reports teaching fees from the Association of British Pharmaceutical Industry (ABPI). Rebecca J. Hoyle is a member of the Scientific Board of Smith Institute. The other authors declare no conflicts of interest.

## Supporting Information

Additional supporting information can be found online in the Supporting Information section.

## Supporting information


**Supporting Information 1** Supporting Information Tables 1–8 detail the early‐life variables considered across five domains, variable missingness, correlations between domain predicted probabilities, and regression coefficients from domain‐specific models examining associations with obesity–hypertension comorbidity.

**Supporting Information 2** STROBE statement: checklist of items that should be included in reports of observational studies.

## Data Availability

The data that support the findings of this study are available from UK Data Service. Restrictions apply to the availability of these data, which were used under license for this study. Data are available from https://ukdataservice.ac.uk/with the permission of UK Data Service.

## References

[1] House of Commons Library , Obesity Statistics, 2023, https://commonslibrary.parliament.uk/research-briefings/sn03336/.

[2] National Health Service Digital , Health Survey for England, Part 2, 2021, https://digital.nhs.uk/data-and-information/publications/statistical/health-survey-for-england/2021-part-2/adult-health-hypertension.

[3] Wolin K. Y. , Carson K. , and Colditz G. A. , Obesity and Cancer, The Oncologist. (2010) 15, 556–565. 10.1634/theoncologist.2009-0285 PMC3227989 20507889

[4] Eckel R. H. , Kahn S. E. , Ferrannini E. et al., Obesity and Type 2 Diabetes: What Can be Unified and What Needs to be Individualized?, Diabetes Care. (2011) 34, no. 6, 1424–1430, 10.2337/dc11-0447. PMC3114323 21602431

[5] Powell-Wiley T. M. , Poirier P. , Burke L. E. et al., Obesity and Cardiovascular Disease: A Scientific Statement From the American Heart Association, Circulation. (2021) 143, no. 21, e984–e1010, 10.1161/cir.0000000000000973. PMC8493650 33882682

[6] Weldegiorgis M. and Woodward M. , The Impact of Hypertension on Chronic Kidney Disease and End-Stage Renal Disease is Greater in Men Than Women: A Systematic Review and Meta-Analysis, BMC Nephrology. (2024) 25, no. 1, 10.1186/s12882-020-02151-7. PMC7687699 33238919

[7] Bhaskaran K. , dos-Santos-Silva I. , Leon D. A. , Douglas I. J. , and Smeeth L. , Association of BMI With Overall and Cause-Specific Mortality: A Population-Based Cohort Study of 3.6 Million Adults in the UK, The Lancet Diabetes & Endocrinology. (2018) 6, no. 12, 944–953, 10.1016/s2213-8587(18)30288-2. PMC6249991 30389323

[8] Zheng Y. , Gao X. , Jia H.-Y. , Li F.-R. , and Ye H. , Influence of Hypertension Duration and Blood Pressure Levels on Cardiovascular Disease and All-Cause Mortality: A Large Prospective Cohort Study, Frontiers in Cardiovascular Medicine. (2022) 9, 10.3389/fcvm.2022.948707. PMC9618611 36324752

[9] World Health Organization , Obesity, 2021, https://www.who.int/news-room/facts-in-pictures/detail/6-facts-on-obesity.

[10] Kario K. , Okura A. , Hoshide S. , Mogi M. et al., The WHO Global Report 2023 on Hypertension: Warning the Emerging Hypertension Burden Worldwide and Its Treatment Strategy, Hypertension Research. (2024) 47, no. 5, 1099–1102, 10.1038/s41440-024-01622-w. 38443614

[11] Leggio M. , Lombardi M. , Caldarone E. et al., The Relationship Between Obesity and Hypertension: An Updated Comprehensive Overview on Vicious Twins, Hypertension Research. (2017) 40, no. 12, 947–963, 10.1038/hr.2017.75. 28978986

[12] Tanaka M. , Improving Obesity and Blood Pressure, Hypertension Research. (2020) 43, no. 2, 79–89, 10.1038/s41440-019-0348-x. 31649313

[13] Shams E. , Kamalumpundi V. , Peterson J. , Gismondi R. A. , Oigman W. , and Correia M. , Highlights of Mechanisms and Treatment of Obesity-Related Hypertension, Journal of Human Hypertension. (2022) 36, no. 9, 785–793, 10.1038/s41371-021-00644-y. 35001082

[14] Mokdad A. H. , Ford E. S. , Bowman B. A. et al., Prevalence of Obesity, Diabetes, and Obesity-Related Health Risk Factors, 2001, JAMA. (2003) 289, no. 1, 76–79, 10.1001/jama.289.1.76. 12503980

[15] Natsis M. , Antza C. , Doundoulakis I. , Stabouli S. , and Kotsis V. , Hypertension in Obesity: Novel Insights, Current Hypertension Reviews. (2020) 16, no. 1, 30–36, 10.2174/1573402115666190415154603. 30987571

[16] Alidu H. , Owiredu W. K. B. A. , Amidu N. et al., Hypertension and Obesity Comorbidities Increase Coronary Risk and Affect Sexual Function and Quality of Life, International Journal of Impotence Research. (2018) 30, no. 1, 8–13, 10.1038/s41443-017-0003-5. 29180795

[17] Stannard S. , Berrington A. , Paranjothy S. et al., A Conceptual Framework for Characterising Lifecourse Determinants of Multiple Long-Term Condition Multimorbidity, Journal of Multimorbidity and Comorbidity. (2023) 13, 10.1177/26335565231193951. PMC10478563 37674536

[18] Alwan N. A. , Stannard S. , Berrington A. et al., Risk Factors for Ill Health: How do We Specify What is Modifiable?, PLOS Global Public Health. (2024) 4, no. 3, 10.1371/journal.pgph.0002887. PMC10911600 38437177

[19] Stannard S. , Berrington A. , Fraser S. D. S. et al., Mapping Domains of Early Life Determinants of Future Multimorbidity Across Three UK Longitudinal Cohort Studies, Scientific Reports. (2024) 14, no. 1, 10.1038/s41598-024-72275-5. PMC11399113 39271760

[20] Stannard S. , Owen R. K. , Berrington A. et al., Socioeconomic, Educational, Family, Perinatal and Developmental Early Life Domains as Predictors of Obesity and Hypertension Comorbidity: Findings From the 1970 British Cohort Study (BCS70), Critical Public Health. (2026) 36, no. 1, 10.1080/09581596.2026.2659481.

[21] Broom M. , Corlett A. , Hale S. , McCurdy C. , and Pacitti C. , An Intergenerational Audit for the UK, 2023, Resolution Foundation.

[22] Gomulka J. and Stern N. , The Employment of Married Women in the United Kingdom, 1970–83, Economica. (1990) 57, no. 226, 171–199, 10.2307/2554159.

[23] Worth E. , Women, Education and Social Mobility in Britain During the Long 1970s, Cultural and Social History. (2019) 16, no. 1, 67–83, 10.1080/14780038.2019.1574052.

[24] Mayhew K. , Deer C. , and Dua M. , The Move to Mass Higher Education in the UK: Many Questions and Some Answers, Oxford Review of Education. (2004) 30, no. 1, 65–82, 10.1080/0305498042000190069.

[25] Allen R. C. , Technical Change, Globalisation and the Labour Market: British and American Experience Since 1620, 2021, https://www.ifs.org.uk/inequality/the-interplay-among-wages-technology.

[26] Fraser S. D. S. , Stannard S. , Holland E. et al., Multidisciplinary Ecosystem to Study Lifecourse Determinants and Prevention of Early-Onset Burdensome Multimorbidity (MELD-B): Protocol for a Research Collaboration, Journal of Multimorbidity and Comorbidity. (2023) 13, 10.1177/26335565231204544. PMC10521301 37766757

[27] Power C. and Elliott J. , Cohort Profile: 1958 British Birth Cohort (National Child Development Study), International Journal of Epidemiology. (2006) 35, no. 1, 34–41, 10.1093/ije/dyi183. 16155052

[28] Little R. J. A. and Rubin D. B. , Statistical Analysis With Missing Data, 2002, 2nd edition, Wiley.

[29] Horton N. J. and Lipsitz S. R. , Multiple Imputation in Practice: Comparison of Software Packages for Regression Models With Missing Variables, The American Statistician. (2001) 55, 244–254.

[30] Jakobsen J. C. , Gluud C. , Wetterslev J. , and Winkel P. , When and How Should Multiple Imputation be Used for Handling Missing Data in Randomised Clinical Trials: A Practical Guide With Flowcharts, BMC Medical Research Methodology. (2017) 17, no. 1, 10.1186/s12874-017-0442-1. PMC5717805 29207961

[31] Collins L. M. , Schafer J. L. , and Kam C. M. , A Comparison of Inclusive and Restrictive Strategies in Modern Missing Data Procedures, Psychological Methods. (2001) 6, no. 4, 330–351, 10.1037/1082-989x.6.4.330. 11778676

[32] Mostafa T. , Narayanan M. , and Pongiglione B. , Improving the Plausibility of the Missing at Random Assumption in the 1958 British Birth Cohort (CLS Working Paper 2020/6), 2020, Centre for Longitudinal Studies.

[33] Centre for Longitudinal Studies , National Child Development Study: Ethical Review and Consent, 2017, https://cls.ucl.ac.uk.

[34] Haapanen M. , Vetrano D. , Mikkola T. et al., Early Growth, Stress, and Socioeconomic Factors as Predictors of the Rate of Multimorbidity Accumulation Across the Life Course, The Lancet. Healthy Longevity. (2023) 5, e56–e65. 10.1016/S2666-7568(23)00231-3 38103563

[35] Harper S. , Lynch J. , and Smith G. D. , Social Determinants and the Decline of Cardiovascular Diseases: Understanding the Links, Annual Review of Public Health. (2011) 32, no. 1, 39–69, 10.1146/annurev-publhealth-031210-101234. 21219168

[36] Humphreys J. , Jameson K. , Cooper C. , and Dennison E. , Early-Life Predictors of Future Multimorbidity: Results From the Hertfordshire Cohort, Age and Ageing. (2018) 47, no. 3, 474–478, 10.1093/ageing/afy005. PMC5935230 29438452

[37] Johnston M. C. , Black C. , Mercer S. W. , Prescott G. J. , and Crilly M. A. , Impact of Educational Attainment on the Association Between Social Class at Birth and Multimorbidity in Middle Age, BMJ Open. (2019) 9, no. 1, 10.1136/bmjopen-2018-024048. PMC6352766 30696675

[38] Gondek D. , Bann D. , Brown M. , Hamer M. , Sullivan A. , and Ploubidis G. B. , Prevalence and Early-Life Determinants of Mid-Life Multimorbidity, BMC Public Health. (2021) 21, no. 1. 10.1186/s12889-021-11291-w PMC8317357 34315472

[39] Cohen S. , Janicki-Deverts D. , Chen E. , and Matthews K. A. , Childhood Socioeconomic Status and Adult Health, Annals of the New York Academy of Sciences. (2010) 1186, no. 1, 37–55, 10.1111/j.1749-6632.2009.05334.x. 20201867

[40] Wu Y. , Gnanapragasam S. , Sanchez-Niubo A. et al., Childhood Socioeconomic Position and Healthy Ageing, BMJ Public Health. (2025) 3, 10.1136/bmjph-2024-001590. PMC11865732 40017978

[41] Furstenberg F. F. , Fifty Years of Family Change: From Consensus to Complexity, The Annals of the American Academy of Political and Social Science. (2014) 654, no. 1, 12–30, 10.1177/0002716214524521.

[42] Kalmijn M. , Mortelmans D. , Feeling of Guilt in the Family: The Case of Divorced Parents, Divorce in Europe, 2020, Springer Open, Berlin, 271–289.

[43] Perelli-Harris B. and Styrc M. , Mental Well-Being Differences in Cohabitation and Marriage, Journal of Marriage and Family. (2017) 80, 239–255. 10.1111/jomf.12431 PMC5811838 29456265

[44] Stannard S. , Berrington A. , and Alwan N. A. , The Mediating Pathways Between Parental Separation in Childhood and Offspring Hypertension at Midlife, Scientific Reports. (2022) 12, no. 1, 10.1038/s41598-022-11007-z. PMC9054745 35488035

[45] Perry S. E. , Fifty Years of Progress in Neonatal and Maternal Transport for Specialty Care, Journal of Obstetric, Gynecologic, and Neonatal Nursing. (2021) 50, no. 6, 774–788, 10.1016/j.jogn.2021.04.013. 34166650

[46] Nuttall F. Q. , Body Mass Index: Obesity, BMI, and Health, Nutrition Today. (2015) 50, no. 3, 117–128. 10.1097/NT.0000000000000092 PMC4890841 27340299

[47] Holland E. , Matthews K. , Macdonald S. et al., The Impact of Living With Multiple Long-Term Conditions (Multimorbidity) on Everyday Life-A Qualitative Evidence Synthesis, BMC Public Health. (2024) 24, no. 1, 10.1186/s12889-024-20763-8. PMC11654051 39696210

[48] Pedersen A. B. , Mikkelsen E. M. , Cronin-Fenton D. et al., Missing Data and Multiple Imputation in Clinical Epidemiological Research, Clinical Epidemiology. (2017) 9, 157–166, 10.2147/clep.s129785. PMC5358992 28352203

[49] Cheng W. , Du Y. , Zhang Q. et al., Age-Related Changes in the Risk of High Blood Pressure, Frontiers in Cardiovascular Medicine. (2022) 9, 10.3389/fcvm.2022.939103. PMC9521719 36187016

[50] Dietz W. , The Science, Strengths, and Limitations of Body Mass Index, Translating Knowledge of Foundational Drivers of Obesity Into Practice, 2022, National Academies of Sciences, Engineering, and Medicine, Washington, DC.

[51] Rubino F. , Cummings D. E. , Eckel R. H. et al., Definition and Diagnostic Criteria of Clinical Obesity, The Lancet Diabetes & Endocrinology. (2025) 13, no. 3, 221–262, 10.1016/S2213-8587(24)00316-4. PMC11870235 39824205

[52] Silveira E. A. , Mendonça C. R. , Delpino F. M. et al., Sedentary Behaviour, Physical Inactivity, Abdominal Obesity and Obesity in Adults and Older Adults, Clinical Nutrition ESPEN. (2022) 50, 63–73, 10.1016/j.clnesp.2022.06.001. 35871953

[53] Hardy R. and Howe L. D. , Mishra G. D. , Hardy R. , and Kuh D. , A Life Course Approach to Body Weight, A Life Course Approach to Women’s Health, 2023, 2nd edition, Oxford University Press, Oxford, 273–292, 10.1093/oso/9780192864642.003.0016.

[54] Altman D. G. and Andersen P. K. , Bootstrap Investigation of the Stability of a Cox Regression Model, Statistics in Medicine. (1989) 8, no. 7, 771–783, 10.1002/sim.4780080702. 2672226

[55] Derksen S. and Keselman H. J. , Backward, Forward and Stepwise Automated Subset Selection Algorithms, British Journal of Mathematical and Statistical Psychology. (1992) 45, 265–282.

[56] Ribeiro D. , Stepwise Selection Algorithms Almost Always Ruin Statistical Estimates, 2025, https://diogoribeiro7.github.io/statistics/stepwise_selection/.

[57] Royston P. and Sauerbrei W. , Multivariable Model-Building, 2008, Wiley.

[58] Lu H. , Cole S. , Platt R. , and Schisterman E. , Revisiting Overadjustment Bias, Epidemiology. (2021) 32, no. 5, e22–e23, 10.1097/ede.0000000000001377. 34172691

[59] Stannard S. , Berrington A. , Fraser S. D. S. et al., Domains of Early-Life Risk and Obesity-Hypertension Comorbidity, Journal of Epidemiology & Community Health. (2024) 78.

